# Progress in the seasonal variations of blood lipids: a mini-review

**DOI:** 10.1186/s12944-020-01237-3

**Published:** 2020-05-25

**Authors:** Xiaochun Ma, Haichen Yan, Haizhou Zhang, Mansen Wang, Qunye Zhang, Xiaoming Zhou

**Affiliations:** 1 Department of Cardiovascular Surgery, Shandong Provincial Hospital affiliated to Shandong University, No.324 Jingwu Road, Jinan, 250021 Shandong China; 2 School of Medicine, Shandong University, No.44 Wenhua Xi Road, Jinan, 250012 Shandong China; 3 Medical Data Research Center, Providence Health & Services, 9205 SW Barnes Road, Suite LL#33, Portland, Oregon 97225 USA; 4 Qilu Hospital of Shandong University, No.107 Wenhua Xi Road, Jinan, 250012 Shandong China; 5 Division of Endocrinology and Metabolism, Shandong Provincial Hospital affiliated to Shandong University, No.324 Jingwu Road, Jinan, 250021 Shandong China

**Keywords:** Seasonal variations of blood lipids, Dyslipidemia, Cholesterol, Hyperlipidemia, Lipid-lowering therapy

## Abstract

The seasonal variations of blood lipids have recently gained increasing interest in this field of lipid metabolism. Elucidating the seasonal patterns of blood lipids is particularly helpful for the prevention and treatment of cardiovascular and cerebrovascular diseases. However, the previous results remain controversial and the underlying mechanisms are still unclear. This mini-review is focused on summarizing the literature relevant to the seasonal variability of blood lipid parameters, as well as on discussing its significance in clinical diagnoses and management decisions.

## Introduction

In the last decades, the cardiovascular and cerebrovascular diseases have become the most prevalent causes of mortality and morbidity worldwide [1]. Amongst all the potential risk factors, dyslipidemia has long been recognized to play a crucial role in the initiation and development of cardio-cerebrovascular diseases [2, 3]. The seasonal variations of blood lipids might be a vital contributor to the seasonal alterations in the mortality and morbidity of these diseases [4]. An array of studies have reported the seasonal changes in the levels of plasma or serum lipids [5–7], which has also been described by the European Atherosclerosis Society (EAS) and the European Society of Cardiology (ESC) in their joint dyslipidemia guidelines [8]. The evaluation of seasonal variations in the lipid profile will be (Delete: is hopefully) considered as a necessity in stratifying risk profiles and identifying high-risk population in near future. However, the seasonal dynamics of various lipid parameters, including triglyceride (TG), cholesterol, low-density lipoprotein cholesterol (LDL-C) and high-density lipoprotein cholesterol (HDL-C), are still undetermined [9–11]. In this mini-review, we collected the relevant literature and summarized the current knowledge about the dynamic changes of LDL-C, HDL-C, TG and cholesterol through the seasons. We also sought to discuss the seasonal effects of lipids on routine clinical practice (Table 1).

**Table 1:** Summary of the included literature

Articles	Refs	Years	Results (Summer)	Results (Winter)
Kreindl C	7	2014	LDL: ↓; HDL: ↑	LDL: ↑; HDL: ↓
Moura FA	12	2013	LDL: ↓; HDL: ↑	LDL: ↑; HDL: ↓
Zhou X	6	2016	M: LDL: ↓; HDL: ↓; Cholesterol: ↓; Triglyceride: ↑ F: LDL: ↑; HDL: ↓; Cholesterol: ↑; Triglyceride: n.s.	M: LDL: ↑; HDL: ↑; Cholesterol: ↑; Triglyceride: ↓ F: LDL: ↓; HDL: ↑; Cholesterol: ↓; Triglyceride: n.s.
Hadaegh F	13	2006	LDL: ↓; Cholesterol: ↓; F: Cholesterol: n.s.	LDL: ↑; Cholesterol: ↑; F: Cholesterol: n.s.
Joshi P	14	2014	LDL: ↓	LDL: ↑
Hopstock LA	15	2013	HDL: ↓	HDL: ↑
Chen SH	16	2006	HDL: ↓; Triglyceride: ↑	HDL: ↑; Triglyceride: ↓
Sasaki J	17	1983	LDL: ↓ (healthy men); HDL: ↓ (healthy men and schizophrenic patients)	LDL: ↑ (healthy men); HDL: ↑ (healthy men and schizophrenic patients)
Letellier G	18	1982	LDL: n.s.; HDL: n.s.	LDL: n.s.; HDL: n.s.
Tung P	19	2009	LDL: n.s.; HDL: n.s.	LDL: n.s.; HDL: n.s.
Fyfe T	9	1968	Cholesterol: ↓	Cholesterol: ↑
Thomas CB	20	1961	Cholesterol: ↓	Cholesterol: ↑
Fuller JH	21	1974	Cholesterol: n.s.	Cholesterol: n.s.
Bull GM	10	1979	Cholesterol: n.s.	Cholesterol: n.s.
Robinson D	11	1992	Cholesterol: ↓	Cholesterol: ↑
Garde AH	22	2000	Cholesterol: ↓	Cholesterol: ↑
Buxtorf JC	23	1988	Cholesterol: ↓	Cholesterol: ↑
Gordon DJ	24	1987	Cholesterol: ↓	Cholesterol: ↑
Ockene IS	25	2004	Cholesterol: ↓	Cholesterol: ↑
Valencia VE	5	2017	Cholesterol: ↓	Cholesterol: ↑

Footnotes: *F* female, *M* male, *n.s* not significant, *↑* high level or elevated, ↓ low level or decreased

### Publication search and inclusion

Eligible studies were searched in the PubMed database which satisfied the following criteria: (A) published randomized control trials (RCTs) or observational studies in English language; (B) focused on the seasonal variability of blood lipids including LDL-C, HDL-C, cholesterol and triglyceride; (C) availability of data required for analysis. Exclusion criteria were: (a) case reports, editorial comments and review articles; (b) studies currently underway or with insufficient data. Three authors (Xiaochun Ma, Haichen Yan and Xiaoming Zhou) independently searched the PubMed databases for retrieval of eligible studies published from inception to December 2018. The search terms used included the “seasonal variations”, “seasonal pattern”, “seasonal variability”; “seasonal alteration”; “seasonal change”; “seasonal dynamics”; “seasonal effect”; “seasonality”; “seasonal difference”; “seasonal trend”; and “seasonal transformation” of blood lipids. The three corresponding authors also manually examined the references from retrieved articles for articles meeting the criteria. The abstracts of retrieved articles were subsequently checked separately by Xiaochun Ma and Haichen Yan. Next, the full-text articles potentially included in this mini-review were finally determined and disagreements were resolved by a further discussion among all the authors. The result was that 20 articles were eventually included in this mini-review and the search flow diagram was shown in the supplementary Fig. 1.

**Fig. 1:**
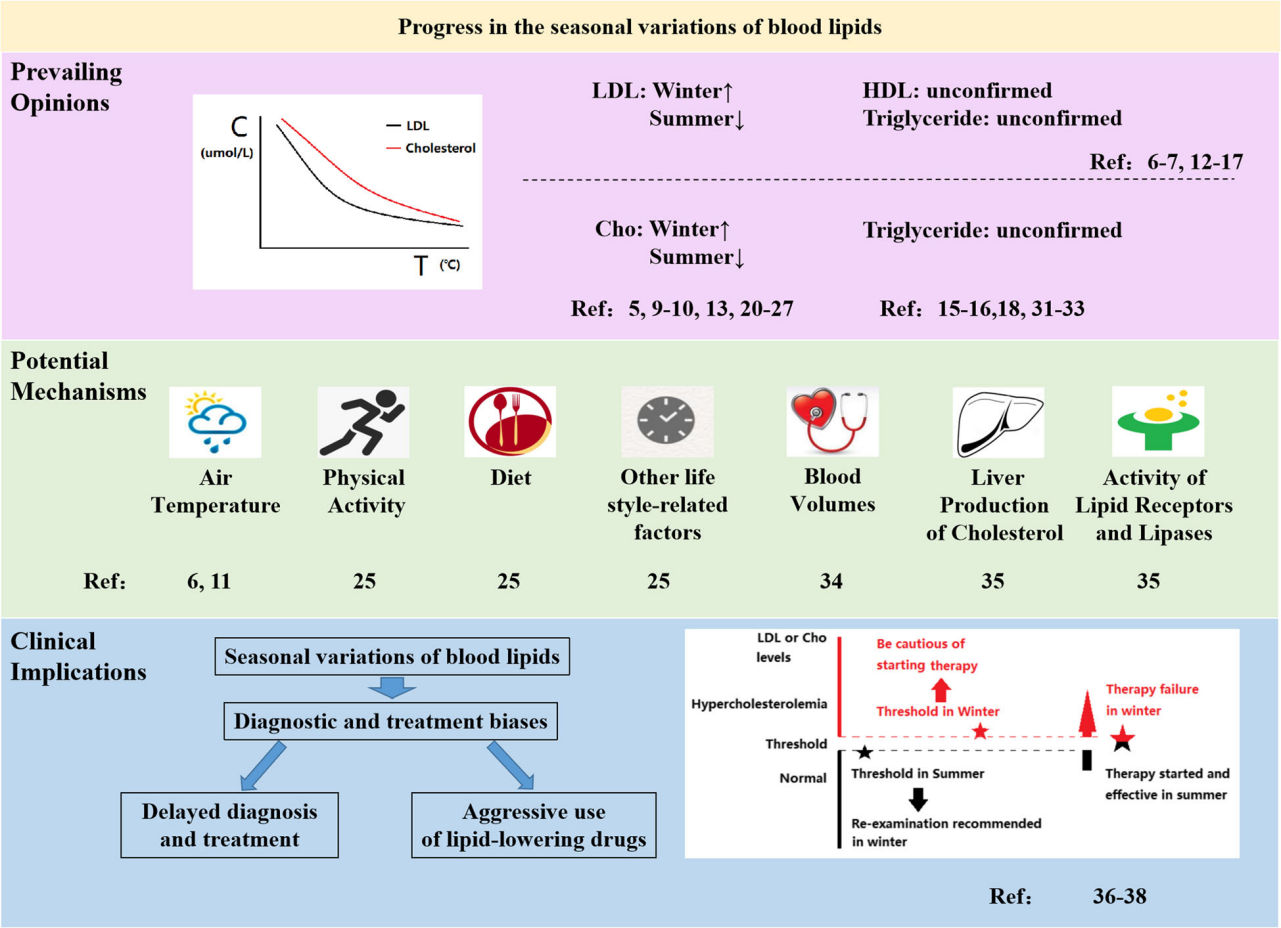
Summary of the prevailing opinions, potential mechanisms and clinical implications for the seasonal differences in blood lipids. 1) Prevailing opinions:.Most reports supports that the levels of LDL-C and cholesterol were elevated in winter and were decreased in summer [6, 7, 12–17]. For HDL-C and triglyceride, the existing results were contradictory [5, 9, 10, 13, 15, 16, 18, 20–27, 31, 32, 38].. 2) Potential mechanisms:.Accumulating evidence pointed towards an array of mechanisms for the seasonal variations of serum lipids, including air temperature, physical activity, diet, blood volume, liver production of cholesterol and activity of lipid receptors and lipases, as well as other life style-related factors [6, 11, 25, 33, 34].. 3) Clinical implications: The seasonal variations of serum lipids could lead to the diagnostic and treatment biases, which include the delayed diagnosis and treatment as well as the aggressive use of lipid-lowering therapy. Firstly, for the individuals whose lipid measures approach or reach the lower limit for therapy in the summer, another measurement is highly recommended in the winter. Otherwise, a fraction of this group might experience a missed diagnosis and delayed treatment. Secondly, for the subjects whose lipid levels just reach or are slightly above the lower limit for therapy in the winter, there might be a bias toward improperly classifying a portion of this group as hypercholesterolemic in the winter. Thirdly, a higher possibility of treatment failure is expected when patients initiate treatment for hypercholesterolemia during the summer and undergo follow-up during the winter [35–37].

### Seasonal variations in low-density lipoprotein cholesterol and high-density lipoprotein cholesterol

The most prevailing viewpoints support the seasonal variations in the serum or plasma levels of HDL-C and LDL-C. However, those results regarding the seasonal patterns of HDL-C and LDL-C were contradictory to a large extent.

Kreindl and his team conducted a follow-up study in which the serum lipid profiles were measured in 50 volunteers for 1 year at monthly intervals. Their findings demonstrated that the levels of serum LDL-C were significantly higher in winter and spring than in summer and fall (*p* < 0.01) whereas the HDL levels were decreased markedly in winter (*p* < 0.05) [7].

Another study by Moura investigated 227,359 consecutive individuals who underwent health checkups in primary care centers between 2008 and 2010. The results showed that the prevalence of LDL-C>130 mg/dl was 8% higher in winter than in summer (*p* < 0.001). And the difference was even larger amongst females and middle-aged individuals (*p* < 0.001). Besides, HDL-C<40 mg/dl were 9% more prevalent in summer (*p* < 0.001). The variation amplitude of HDL-C was 3.4 ± 0.3 mg/dl and 7.0 ± 2 mg/dl for LDL-C. This finding presented the evidence of biological rhythms and seasonal variations in lipid profiles based on a large population sample [12].

Our team performed a longitudinal study using health examination data of 5 consecutive years (47,270 subjects) in Jinan, China. It was pointed out that for males both the levels of HDL-C and LDL-C decreased significantly for every 10 °C increase in air temperature (HDL-C: 0.18 mmol/L and LDL-C: 0.06 mmol/L). The values of LDL-C might therefore reach its peak in winter. For females, there was an increase in the levels of LDL-C (0.26 mmol/L) and a decrease in the levels of HDL-C (0.32 mmol/L) for every 10 °C increase. Those classical lipid-associated risk factors were adjusted before reaching the conclusions, such as age, gender, diet, exercise, blood pressure, body weight, change of body weight, body mass index, glycemia, alanine aminotransferase and creatinine. Thus air temperature might represent an independent influencing factor for LDL-C and HDL-C levels and men and women are distinctly affected [6].

The same issue has been addressed to explore the seasonal variability of serum lipids in adults in Tehran Lipid and Glucose Study. It was a cross-sectional study recruiting 2890 men and 4004 women aged 20 to 64 years from the Tehran Center for Fat and Blood Glucose Study (TLGS) between 1999 and 2001. The LDL-C and HDL-C values varied significantly in different seasons in males, with higher LDL-C values in winter than in summer (*p* < 0.05). In females, the values of LDL-C and HDL-C were consistent regardless of seasonality. The prevalence of high LDL-C (>or = 160 mg/dl) was 26.7 and 24.9% respectively in men and women in winter, which increased dramatically in both genders in winter (*p* < 0.05). Interestingly, this study concluded that the seasonal variability in serum lipid values might be greater in men than in women. And the elevated prevalence of high LDL in winter in both sexes should be taken into consideration in the prevention and treatment of cardio-cerebrovascular diseases [13].

Another large-scale study by Joshi and his team analyzed the seasonal variations of blood lipoproteins in 2859,333 consecutive American adults recruited from 2006 to 2013 [14]. Particularly, the seasonal changes in blood lipids were compared, as were the incidences of abnormal LDL-C and HDL-C according to guidelines-based goal attainment. Men and Women varied by about 2 and 4 mg/dl for LDL-C and HDL-C, respectively. Similarly, the levels of LDL-C peaked in winter and it might lead to a variety of pathophysiological changes. However, the authors concluded that the significance of clinical guidance was not sufficient [14].

Hopstock observed 38,037 participants in a population-based cohort from 1979 to 2008 in regards to seasonal variations in the cardiovascular disease risk factors and LDL-C were also on the list [15]. And a community-based study was conducted to explore the summer-winter differences of component of metabolic syndrome and included a total of 8251 residents aged 40 in Kinmen, Taiwan [16]. These two studies both indicated a peak time in winter of HDL-C levels.

Sasaki et.al. studied the seasonal variations in serum lipoproteins in schizophrenia patients and healthy subjects. Their report demonstrated that the HDL-C levels in summer and autumn were significantly lower than those in winter and spring in both healthy men and schizophrenic patients. And the concentration of LDL-C was significantly higher in September and October as compared with April in healthy men [17].

However, Letellier and his colleagues performed a 4-year study of 2600 female individuals aged 30 to 39 years and were unable to confirm the variations previously reported [18]. Tung and his team re-examined the data from the PROVE IT-TIMI 22 study and also detected no significant difference in LDL-C when stratified by season [19].

### Seasonal variations of cholesterol

Studies on the seasonal variations of cholesterol have been traced back to early decades. In 1960s, cholesterol levels have been showed significantly higher in winter than in summer in the general population [9, 20]. In 1971, Fuller with his team conducted an epidemiological survey on 1005 healthy subjects and the effects of seasonality on cholesterol and triglyceride levels were investigated [21]. Nevertheless, no obvious changes in the cholesterol levels was found (deleted: noticed) between different seasons in their studies. Subsequently, Bull and his colleagues also obtained a consistent conclusion in his investigation [10].

Since then a large quantity of studies have been performed to explore the seasonal variations in cholesterol and the most results advocated a higher level of cholesterol in winter than in summer. Robinson et.al. performed spectral analyses spanning over 4 years which included 140,000 men and 32,000 women in the UK and 30,000 men and 12,000 women in Japan respectively. The results demonstrated a strong seasonal effect on the serum cholesterol levels, with average cholesterol levels about 3–5% higher in winter than in summer. Besides, monthly mean cholesterol level was negatively correlated with monthly mean temperature. And the seasonal differences in cholesterol levels were independent of body weight and other potential confounding factors. It was the first study on the seasonal variation of cholesterol and the results were based on two large populations which include more than 200,000 subjects. The findings might have important implications for both long-term epidemiological or follow-up studies as well as for the interpretation of patient data [11]. The conclusions from Robinson’s study have been subsequently confirmed by a series of other studies [22–24].

A 12-month longitudinal study of seasonal variations in blood lipids collected 517 healthy volunteers from a health maintenance organization serving central Massachusetts [25]. This study confirms the seasonal variations in the cholesterol levels both in men and women, with a peak in winter. And the seasonal variations were greater in women and hypercholesterolemic individuals [25].

Hadaegh’s study also compared the levels of serum cholesterol in different seasons. There existed a significant trend for change in the values of cholesterol in men, with higher cholesterol in winter than in summer. The prevalence of hypercholesterolemia in the winter was 26.2% higher than in summer in men. However, the values of cholesterol were similar in women in different seasons, suggesting a gender difference in seasonal variability of serum cholesterol levels [13].

Jerzy with his team collected one-year data of serum cholesterol (304,156 data points) from the laboratories in 14 districts in Poland. JEG, a primitive statistical method (http://ibib.waw.pl/JEGen.html), was used to give three levels of the Gaussian portion of any analytic dataset (Gauss reference interval, GRI): low (GRImin), modal or intermediate (GRIopt) and high (GRI-max). Their results demonstrated the seasonal variability of cholesterol levels in any analyzed subpopulation [26, 27].

Our research also pointed to the seasonal variations of total cholesterol which decreased significantly for every 10 °C increase in air temperature (0.35 mmol/L for men and 0.73 mmol/L for women) [6]. From a mechanistic point of view, seasonal variations in the lipid profile might be explained by a set of seasonal alterations such as hemodilution during the summer and hemoconcentration in winter, as well as changes in dietary behaviors and physical activities between different seasons [25, 28, 29]. We proposed that the annual air temperature fluctuations might be an important mechanism of the seasonal changes of lipids.

Valencia and his team conducted a cross-sectional study of 3366 patients from January 2013 to December 2013. The average level of cholesterol was 180.62 mg/mL in winter, 181.00 mg/mL in spring, 174.90 mg/mL in summer and 179.22 mg/mL in autumn. The prevalence of cholesterol level above the recommended limit was 24.86% in summer, 28.40% in autumn, 31.43% in winter and 31.86% in spring [5].

### Seasonal variations in triglyceride

The studies on the seasonal variations of TG have produced conflicting results with no consensus at present. More intensive investigation is required to resolve this issue. Although a prevailing view supports the levels of TG characteristic of seasonal variations, its peak time remains largely undermined.

In Chen’s study summer was positively associated with the occurrence of hypertriglyceridemia [16]. Our investigation also emphasized that TG levels increased significantly for every 10 °C increase in air temperature (0.12 mmol/L for men and not affected by air temperature amongst women), suggesting a peak time of TG in summer [6]. However, in Hopstock’s survey, TG levels reached its peak in autumn [15].

Letellier’s work showed that for women aged 30 to 39 years, TG was 6% higher in spring than in autumn. Nevertheless, men aged 30 to 39 years had a 22% higher TG in winter than in autumn. The difference was smaller for younger men, and not significant for men aged 20 to 25 years [18].

### Seasonal variations of blood lipids in patients

Most studies above explored the seasonal variations of blood lipids in healthy subjects. Are there seasonal alterations of blood lipids when it comes to patients with basic diseases?

Bardini et.al. investigated the seasonal variability of blood lipids in patients with type 2 diabetes mellitus (DM). The results demonstrated a peak of cholesterol, LDL-C and TG levels in fall and winter, during which HDL-C levels were reduced. To our knowledge, this was the first report showing seasonal changes in lipid profile in type 2 DM outpatients taking or not taking statins. Hence the seasonal variations of lipids might not be exclusive to healthy individuals [30].

Sangha with his team examined the seasonal trends in HbA1c and other metabolic parameters in a cohort of patients with Type 1 DM From 2008 to 2013. It was a retrospective analysis of outpatients attending clinic at a large tertiary referral hospital in the UK. At each clinic visit, HbA1c and other metabolic parameters including cholesterol, TG, creatinine, blood pressure and body mass index were measured. Their findings showed no significant seasonal effect on the metabolic parameters except for HbA1c [31].

A Japanese study investigating a total of 1202 patients with metabolic syndrome showed that serum levels of HDL-C was significantly higher in winter than in summer. This result directly supported to the notion that seasonal variation in metabolic parameters affects the diagnosis of metabolic syndrome [31].

Besides, a study on the seasonal variations of serum lipids in renal transplant recipients (RTR) was performed because this group of patients were at high risk for hyperlipidemia. Their results found that RTR do not exhibit seasonal variations in lipids, unlike the general population. The seasonal effects might be suppressed by the factors unique to RTR such as immunosuppressive therapies etc. [32]

### The significance of seasonal variations of serum lipids in clinical practice

Despite that for decades numerous studies have reported the seasonal variations of serum lipids, the mechanisms of seasonal changes in the metabolism of lipids are not fully determined. And there exist considerable concerns about the consistency and validity of those findings. Besides, the seasons of serum lipid measurements should be given consideration in the diagnosis and management decisions in clinical practice (Additional file 1).

The changes in air temperature have been noted to contribute to the seasonal variations in lipid levels. Robinson and our work both reported the negative correlation of air temperature and cholesterol levels [6, 11]. Additionally, seasonal variations in physical activity, diet and other life style-related factors appeared to explain a small fraction of the observed seasonal variations in blood lipid levels [25]. Importantly, these potential covariates, especially temperature and physical activity, might synergistically lead to the alterations in the blood volumes, an effect further resulting in a decrease of cholesterol levels in summer as a consequence of hypervolemia and an increased level in winter due to hemoconcentration [33]. Besides, the seasonal variabilities of serum lipids might also be attributed from the seasonal alterations in the liver production of cholesterol as well as activity of lipid receptors and lipases [34].

In routine clinical practice, measuring the serum cholesterol levels is crucial in establishing the diagnosis of hypercholesterolemia and evaluating the efficacy outcomes of lipid-lowering therapy. Thanks to the seasonal alterations of serum lipid profiles, difference in the prevalence of hypercholesterolemia in different seasons could produce misclassification for hypercholesterolemia. And this misclassifcation unavoidably leads to diagnostic and treatment biases [29]. In one respect, the bias is connected with the delayed onset of therapeutic intervention and an augmented risk of cardiovascular and cerebrovascular events. In another aspect, aggressive administration of lipid-lowering drugs might occur and result in unnecessary medical expense, adverse reaction and drug toxicity. This raises the concern about adjusting the diagnosis criterion of hypercholesterolemia and therapy stragety according to the seasonal transformation. However, no consensus has been to date reached, nor has any guidelines clearly addressed this issue. Additionally, an interesting question might also be put forward: is it the actual serum lipid concentration or absolute amount of cholesterol carried in the circulatory system that is the genuine culprit in hypercholesterolemia? This question awaits for further elucidation and season-specific guidelines have to be adjusted in the future [25].

Based on the prevailing viewpoint of seasonal variations in the levels of serum cholesterol, we summarized several point views of lipid management (delete: reminders) from the clinician’s perspective. These opinions should be interpreted cautiously because the necessity of clinical intervention due to the seasonal changes of blood lipids is still undetermined given the small and heterogeneous amount of evidence. Firstly, for the individuals whose lipid measures approach or reach the lower limit for therapy in the summer, another measurement is highly recommended in the winter. Otherwise, a fraction of this group might experience a missed diagnosis and delayed treatment. One study suggested that the subjects who are obese, current smoker, advanced in age or carring other well-documented risk factors should particularly consider a repeated serum lipid test in winter [35]. Secondly, for the subjects whose lipid levels just reach or are slightly above the lower limit for therapy in the winter, there might be a bias toward improperly classifying a portion of this group as hypercholesterolemic in the winter. Additionally, more individuals are subject to the diagnostic and treatment selection biases in winter because more adverse cardiovascular and cerebrovascular events occur during the winter months. One team estimated that this misclassification of cholesterol status due to seasonal variation might result in approximately 800 thousand people likely being administrated for hypercholesterolemia in winter at a cost of 840 million dollars in drug treatment alone in the United States [36]. The expense would be huge for the developing countries, needless to say the adverse reaction and drug toxicity from aggressive therapy. Thus for this group, the advent of treatment should be cautious by comprehensively assessing the risk of adverse events and benefit of therapy. Thirdly, a higher possibility of treatment failure is expected when patients initiate treatment for hypercholesterolemia during the summer and undergo follow-up during the winter. It is still not evidence-based that whether or not the lipid-lowering therapy should be intensified or maintained with former dosages for this group in winter. And it is worthy of discussion that whether a season-based individual-tailered therapy strategy is better to adapt to the seasonal alterations of cholesterol. For this purpose, it was highlighted to pay more attention to LDL-C levels rather than on total cholesterol levels, in order to decrease the chance of misclassification. It is because the magnitude of order of LDL-C is lower than that of total cholesterol [37].

Writing a systematic review with meta-analysis is definitely a wise option and an ideal way for this topic. However, after article search and quality assessment, it was found impossible to pool the results using meta-analysis and obtain a trustworthy conclusion. The reason lies in the obvious heterogeneity between these included studies as follows: 1) a wide time span from 1968 to 2018; 2) various ethnicities or coutries from Europeans to Asians; 3) different testing methods of blood lipids; 4) distinct study quality from low to high; 5) last but not least, outcomes or parameters not identical for statistical pooling. A simple example is that different ethnicities are different in the diet structures, living environments, education levels, fitness habits and especially genetic backgrounds, which might have huge influences on the blood lipids and lead to potential bias to the meta-analysis. Hence, if these studies are statistically merged for a meta-analysis, the pooling results will be evidently heterogeneous and not be convincing. Besides, subgroup analysis could be applied to resolve the problem of heterogeneity. However, we estimate that there would be too few studies in each subgroup and therefore the results of meta-analysis would be not reliable. In the near future such a meta-analysis of suitable studies might be performed for the seasonality of blood lipid.

## Conclusions

Most current studies advocated that the season might be a fundamental factor in the changes of blood lipids. And the seasonal differences in blood lipids might be most obvious between winter and summer. There is a certainty that subjects with different ages and genders are distinctly affected by seasonality with regards to blood lipids. At present, the detailed seasonal patterns of blood lipids are still controversy. The seasonal increase in LDL and cholesterol levels in winter might potentially and partially account for the increased mortality and morbidity of cardiovascular and cerebrovascular diseases in winter. From the clinical viewpoint, such amplitude of variations in lipid profile must be considered in the disease prevention and treatment. Besides, the mechanisms of the alterations in blood lipids with the seasons have not yet been established. Further studies with higher quality in research design and larger sample sizes, as well as those studies focused on the mechanisms of this seasonal variability (delete: relevant basic researches), are desperately required to resolve these puzzles.

## Supplementary information

**Additional file 1.**

## Data Availability

Not applicable.

## Abbreviations

EAS: European Atherosclerosis Society
ESC: The European Society of Cardiology
TG: triglyceride
LDL-C: Low-density lipoprotein cholesterol
HDL-C: High-density lipoprotein cholesterol
RCTs: Randomized control trials
TLGS: Tehran Center for Fat and Blood Glucose Study
DM: Diabetes mellitus
RTR: Renal transplant recipients
