# Supplementary figures and images for: Progress in the seasonal variations of blood lipids: a mini-review
Source: Lipids Health Dis. 2020 May 25;19:108. doi: 10.1186/s12944-020-01237-3 (PMC7249447; doi:10.1186/s12944-020-01237-3)

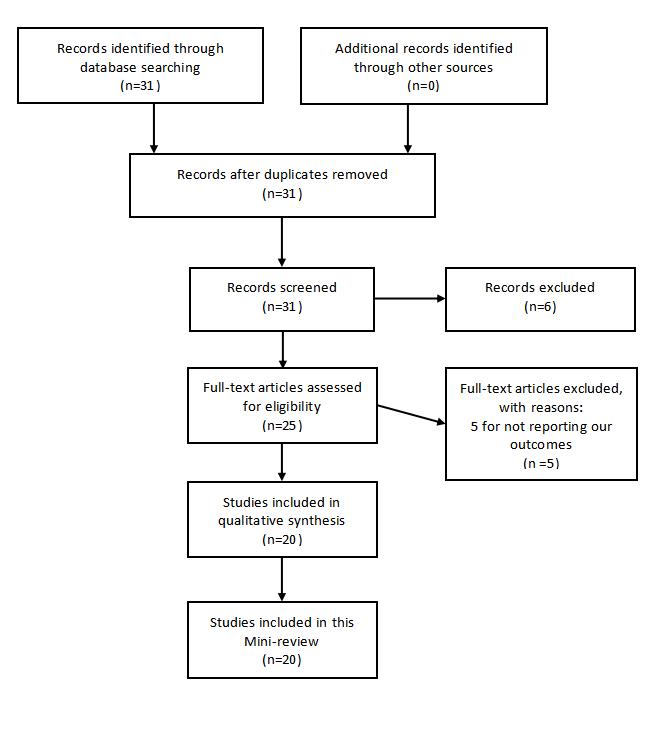

Supplement: Supplementary file 1 — Additional file 1. [file 12944_2020_1237_MOESM1_ESM.jpg]
